# Suspected Brugada Phenocopy Secondary to Coronary Slow Flow

**DOI:** 10.1155/2019/9027029

**Published:** 2019-12-06

**Authors:** Alicia Shim, Rajeev Seecheran, Valmiki Seecheran, Sangeeta Persad, Shiva Sreenivasan, Ronald Henry, Naveen Anand Seecheran

**Affiliations:** ^1^Cardiology Unit, Advanced Cardiovascular Institute, Port of Spain, Trinidad and Tobago; ^2^Department of Medicine, North Central Regional Health Authority, Mt. Hope, Trinidad and Tobago; ^3^School of Medicine, Dentistry, and Biomedical Sciences, Queen's University, Belfast, UK; ^4^Department of Clinical Medical Sciences, University of the West Indies, St. Augustine, Trinidad and Tobago

## Abstract

Brugada syndrome (BrS) is a genetic condition that accentuates the risk of potentially lethal ventricular arrhythmias and sudden cardiac death (SCD) in a structurally normal heart. The Brugada electrocardiographic pattern may manifest separately from the syndrome—this clinical scenario has been described as Brugada phenocopy (BrP). Many etiologies of BrP have been reported, but it has not yet been reported as a result of coronary slow flow (CSF) phenomenon. This case report highlights a suspected coronary slow flow-associated Brugada type 1 electrocardiographic pattern, which subsequently normalized following the institution of guideline-directed medical therapy for acute coronary syndrome.

## 1. Introduction

Brugada syndrome (BrS), initially described in 1992, is a genetic condition with a characteristic electrocardiographic (ECG) pattern and is associated with an elevated risk of life-threatening ventricular arrhythmia (VA) and sudden cardiac death (SCD) [1]. It is typically considered a primary electrical condition without structural heart disease [2]. The ECG pattern comprises ST-segment elevation > 2 mm in one or more leads from V_1_ to V_3_ with “coved-type” descending to inverted T wave or “saddleback” morphology consistent with Brugada type 1 and type 2 patterns, respectively [3, 4]. The prevalence varies internationally and genealogically, typically with a male predilection. Appropriate risk stratification and management of these patients remain a topic of considerable debate [2].

The Brugada type 1 electrocardiographic pattern can manifest separately from the definite syndrome, and this clinical scenario has been called Brugada phenocopy (BrP) [2]. Diagnostic maneuvers (e.g., cardiac sodium channel blockade provocation testing) are often performed to differentiate true BrS from BrP, which can be encountered in a multitude of clinical scenarios and, ultimately, can result potentially in life-saving interventions [5, 6].

Currently, there are no cases reported on BrP observed in patients with coronary slow flow, although it has been recognized in ischemia [7–9]. This report describes a patient with coronary slow flow who presented with a Brugada type 1 ECG pattern, which subsequently normalized following resolution of the endothelial dysfunction.

## 2. Case Report

A fifty-nine-year-old lady of mixed Caribbean-Black and Asian descent with a history of essential hypertension, dyslipidemia, obesity, generalized anxiety disorder, and pharmacological nonadherence presented with chest pain which followed an emotionally stressful encounter (abrupt marital separation). There was no prior history of syncope, presyncope, or palpitation; however, there was a paternal history of sudden cardiac death. Physical examination revealed hypertension but was otherwise normal. Initial routine laboratory results, including cardiac biomarkers, were normal.

**Figure 1:**
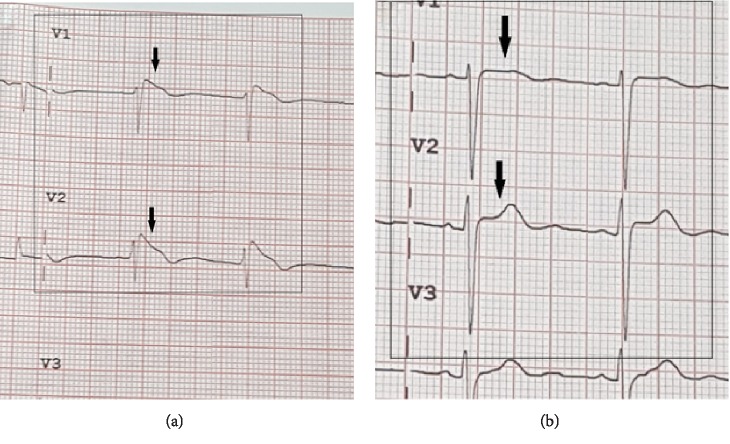
There is the characteristic ST-segment elevation ≥ 2 mm in ≥1 right precordial leads (V_1_ to V_3_), following an *r*′-wave and a straight ST-segment. Additionally, the descending ST-segment crosses the isoelectric line and is followed by a negative and symmetric T wave. At 40 ms of high takeoff, the decrease in amplitude of ST is ≤4 mm and the duration of QRS is longer than that in a right bundle branch block [3]. No high-pass filters were applied to attenuate low-frequency noise [15, 16]. (a) Admission ECG leads V_1_ and V_2_ upon presentation, showing coved ST-segment elevation (black arrows) with elevated J points, suggestive of a Brugada type 1 pattern. (b) Subsequent ECG showing resolution of the Brugada type 1 pattern (black arrows).

The admission electrocardiogram displayed coved ST-segment elevation in V_1_ and V_2_ suggestive of a Brugada type 1 pattern (Figure 1(a)). She was treated with aspirin, ticagrelor, intravenous heparin and nitroglycerin infusions, high-intensity statin, beta-adrenergic and calcium channel blockade, angiotensin-converting enzyme inhibition, and mineralocorticoid receptor antagonist pharmacotherapy. A chest radiograph was unremarkable, with transthoracic echocardiography showing preserved left ventricular function, with no regional wall abnormalities, and grade 1 diastolic dysfunction. Tentative diagnoses for ST-segment elevation at this juncture included aneurysm formation, dissection, hyperkalemia, and pulmonary embolism, which were all effectively excluded with requisite investigations [10].

Coronary angiography revealed mild luminal irregularities with Thrombolysis In Myocardial Infarction (TIMI) 2 antegrade flow suggestive of endothelial dysfunction in the right coronary artery (Supplemental Video Files, [Supplementary-material supplementary-material-1], [Supplementary-material supplementary-material-1]b, and [Supplementary-material supplementary-material-1]). Subsequent ECGs done when pain-free revealed normalization of the Brugada pattern (Figure 1(b)).

Serial measurements of cardiac biomarkers remained normal throughout her inpatient stay, and she was discharged on guideline-directed medical therapy with scheduled outpatient follow-up. The patient subsequently declined further cardiac sodium channel blockade provocation testing and genetic testing for *SCN5A* [11–13]. The patient and her family were counseled with respect to accessing online resources such as the Brugada phenocopy international registry and online educational portal for further insight into her suspected condition [14].

## 3. Discussion

Coronary slow flow (CSF) phenomenon, initially described by Tambe et al. in 1972, is an angiographic entity characterized by delayed progression of the injected contrast medium through the coronary vasculature, typically encountered in patients presenting with clinical features of acute coronary syndrome [17]. It is reported to have an incidence of approximately 7% in patients undergoing coronary angiography. The pathophysiology has not been fully elucidated; however, several structural and mechanistic effects with a resultant maladaptive milieu have been proposed [18, 19]. It has been implicated with life-threatening arrhythmias and sudden cardiac death; however, the most common clinical presentation is that of unstable angina [20, 21]. It is defined by the presence of angiographically normal or near-normal coronary arteries and Thrombolysis In Myocardial Infarction (TIMI) 2 flow (i.e., requiring ≥3 beats to opacify prespecified branch points in the distal vasculature of at least one of the three major epicardial coronary vessels) [22, 23].

The endothelium plays a pivotal role in vasomotor regulation and platelet reactivity and is intimately involved in atherogenesis [18]. Patients with CSF have diffuse intimal thickening and microcalcification along the vessel wall [24] and also tend to have elevated endogenous inflammatory markers such as C-reactive protein and interleukin-6 [25]. Additionally, impaired laminar flow occurs in arterial segments with tortuosity and bifurcations [26]. Clinically, CSF tends to appear in patients with metabolic syndrome—high total cholesterol and low-density lipoprotein cholesterol, elevated fasting glucose, and raised body mass index, all of which were present in our patient [27, 28].

Statin therapy appears beneficial for patients with CSF and is likely to be as a result of their anti-inflammatory properties [29, 30]. Beta-adrenergic blockade can also improve endothelial function and attenuate symptoms by increased nitric oxide release [31]. Our patient's symptoms and ECG appearances quickly resolved after administration of these therapies.

BrP may occur in relation to cardiac sodium (Na) and potassium (K) channel blocking effects or cardiac structural abnormalities, particularly those affecting the right ventricle, such as right ventricular outflow tract ischemia (RVOT) [32]. Occlusion or spasm of the conus branch of the right coronary artery and ischemia of the right ventricular outflow tract and BrP have been previously reported. This has also been replicated in experiments involving acetylcholine, giving credence to the hypothesis that ischemia and vagal influences act additively or synergistically with the substrate responsible for ST-segment elevation in BrS and precipitation of ventricular fibrillation (VF) [33, 34]. It has been postulated that ST-segment elevation occurs maybe due to a decrease in ionized calcium (iCa) caused by acetylcholine or an increase in the adenosine triphosphate- (ATP-) sensitive K channels caused by ischemia resulting in voltage gradients contributing to the manifestation of the Brugada ECG pattern [8, 35]. Furthermore, it has recently been demonstrated that reduced Na channel availability in the ventricular epicardium may contribute to electrical depression and thus may contribute to the ST-segment changes in acute myocardial ischemia [36]. Additionally, in 2015, Agrawal et al. reported BrP during stent implantation distal to the conus branch, which resolved after deployment. They postulated that ischemia of the Purkinje system of the right ventricle might have slowed conduction to the RVOT. Resolution of ischemia restored normal conduction and resolved the ECG pattern. Repolarization abnormalities are less likely to be attributed to this finding because flow reduction was not confined to the conus branch and would have therefore not directly impacted on the RVOT [37]. This case is similar to the one previously described by Peter and further highlights the possible association between RVOT ischemia and BrP [38].

It is difficult to estimate the impact of BrS due to its uncertain prevalence; however, the incidence of the BrS electrocardiographic pattern remains less than 1% in several studies while accounting for approximately 10% of sudden deaths [39]. Implantable cardioverter-defibrillator device therapy is considered the gold standard; however, radiofrequency catheter ablation is rapidly gaining traction as an emerging strategy [2].

Clinical conditions separate from BrS, which electrocardiographically mimic the BrS type 1 ECG pattern, which then resolves with appropriate treatment, have been referred to as Brugada phenocopies [40]. Differentiation between these two entities is critical, as BrP usually represents a more benign prognosticator [14]. The BrP ECG pattern is indistinguishable from that of BrS, and misdiagnosis can have devastating sequelae [41–43]

Our patient's initial ECG was consistent with a Brugada “coved type 1” phenocopy for several reasons, although the presence of a negative P wave component in V_2_ could suggest electrode malposition [2, 4]. There is the characteristic ST-segment elevation ≥ 2 mm in ≥1 right precordial leads (V_1_ to V_3_), following an *r*′-wave and a straight ST-segment. Additionally, the descending ST-segment crosses the isoelectric line and is followed by a negative and symmetric T wave. At 40 ms of high takeoff, the decrease in amplitude of ST is ≤4 mm and the duration of QRS is longer than that in a right bundle branch block [3]. No high-pass filters were applied to attenuate low-frequency noise [15, 16].

Our patient's pretest probability of BrS was determined to be intermediate, as there was no previous history of syncope, palpitations, or witnessed nocturnal agonal respiration; however, there was a positive family history of sudden cardiac death. She declined provocative testing as well as genetic testing for the *SCN5A* gene mutation. Genetic testing, however, is not considered mandatory, as the *SCN5A* mutation is identifiable in only 20% to 30% of probands affected by true BrS [44].

As per the BrP morphologic classification criteria, our patient would potentially be classified as a type 1B BrP (as drug challenge was not performed), which resolved with administration of the acute coronary syndrome and neurohormonal therapies [45].

Differentiating between BrS and BrP is critical because patients with BrS are susceptible to SCD and may require an implantable cardioverter-defibrillator (ICD) with its attendant complications.

## 4. Conclusion

We report the first case of a patient with a suspected Brugada phenocopy thought to be as a result of coronary slow flow and whose ECG reverted to normal following institution of guideline-directed medical therapy.

## Additional Points

*Key Clinical Message*. Be cognizant that coronary slow flow may induce a transient, reversible Brugada-type electrocardiographic pattern (phenocopy), which normalizes upon resolution of the underlying endothelial dysfunction.

## Supplementary Materials

Supplementary 1 Cineangiography of the RCA. Video 2a: left anterior oblique (LAO) view indicating the “slow flow” phenomenon, especially within the acute right ventricular marginal subbranch.

Supplementary 2 Video 2b: right anterior oblique (RAO) view indicating the “slow flow” phenomenon, with complete opacification of the entire vessel taking more than three beats.

Supplementary 3 Video 2c: straight cranial view indicating the “slow flow” phenomenon with delayed contrast media clearance after injection.
